# Supplementary material for: Dysfunction of an Anaphase-Promoting Complex Subunit 8 Homolog Leads to Super-Short Petioles and Enlarged Petiole Angles in Soybean
Source: Int J Mol Sci. 2023 Jul 3;24(13):11024. doi: 10.3390/ijms241311024 (PMC10342176; doi:10.3390/ijms241311024)
Supplement: Supplementary file 1 [file ijms-24-11024-s001.zip › ijms-2437205-supplementary.pdf]

Supplementary Files

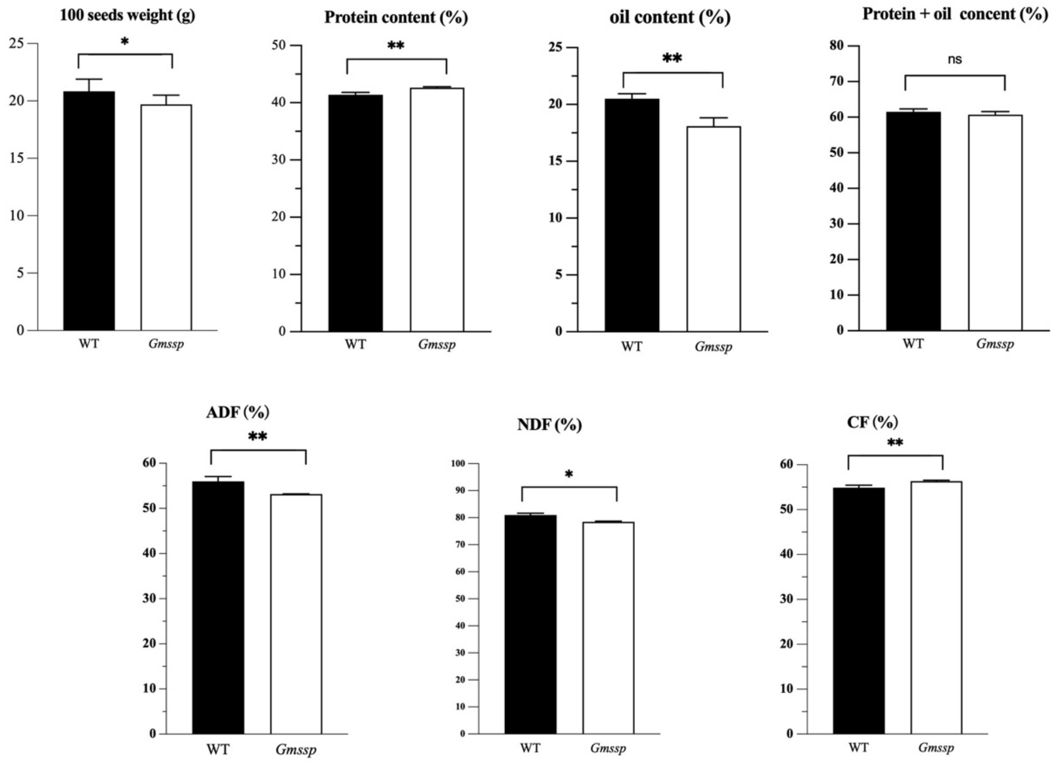

Figure S1. Some phenotypic and physiological traits of *Gmssp* mutant in comparison with wild type in soybean. These traits included 100 seeds weight, protein content, oil content, Protein + oil content, neutral detergent fiber (NDF), acid detergent fiber (ADF), crude fiber (CF). *, ** significant differences at $p < 0.05$ and $p < 0.01$, respectively; ns, not significant.

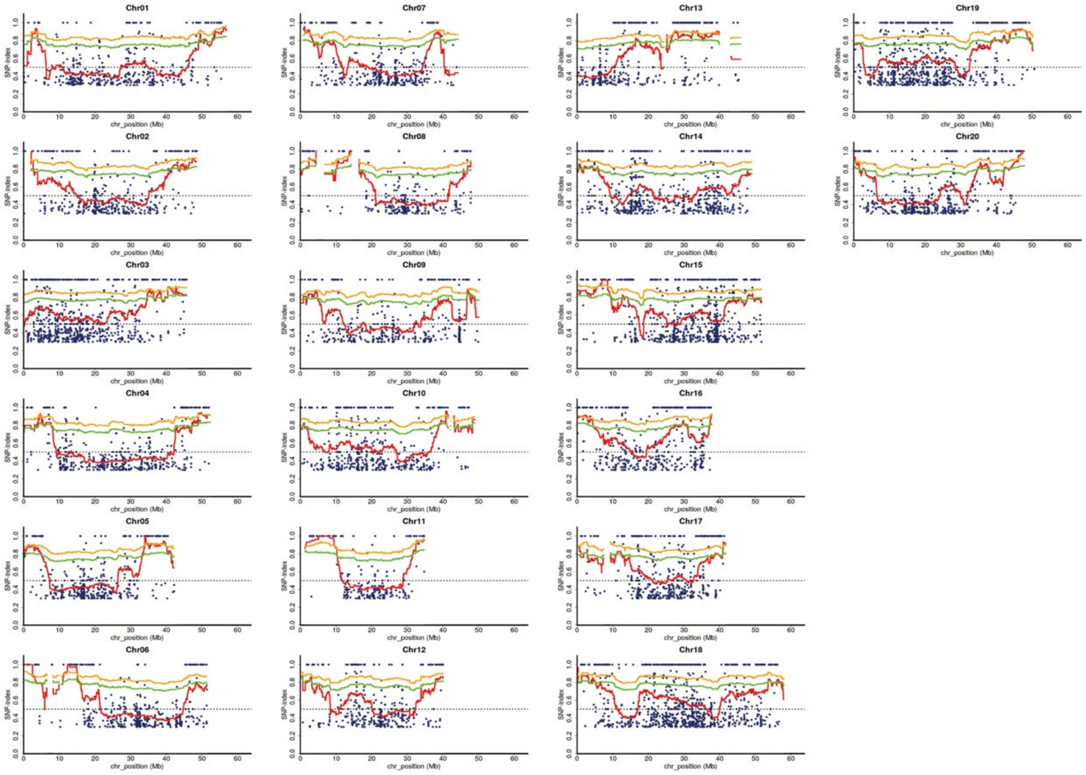

Figure S2. Identification of causal mutations for *Gmssp* using MutMap. The red line showing sliding window average of SNP-index; the green lines showing sliding window average of 95%-confidence interval upper side; the orange line showing sliding window average of 99%-confidence interval upper side. Default parameters were followed to the manual for Version of 1.4.4Rev.0.0 of MutMap_protocol.

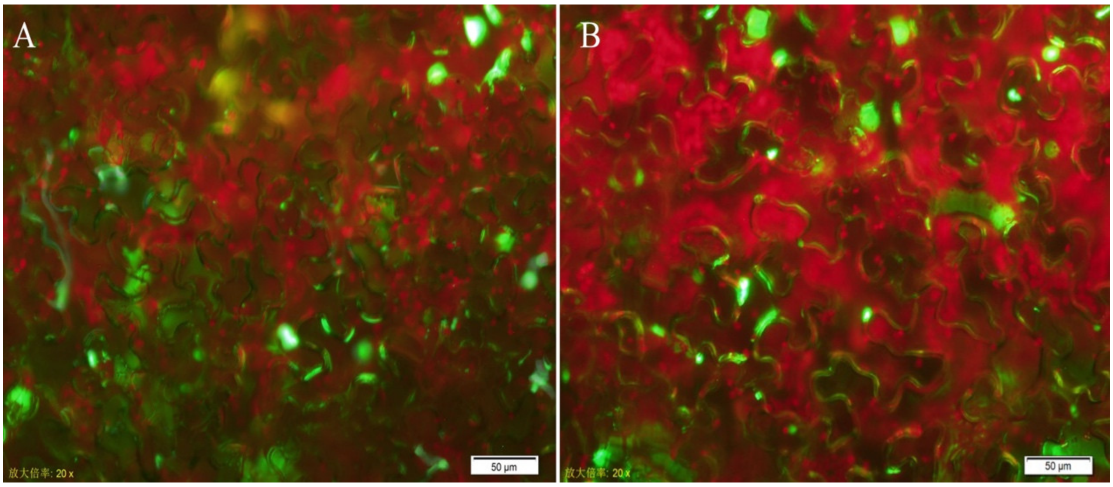

Figure S3. Subcellular localization analysis of GmSSP and Gmssp protein in lower epidermal cells of tobacco leaves. (A) GmSSP-gfp vector; (B) Gmssp-gfp vector.

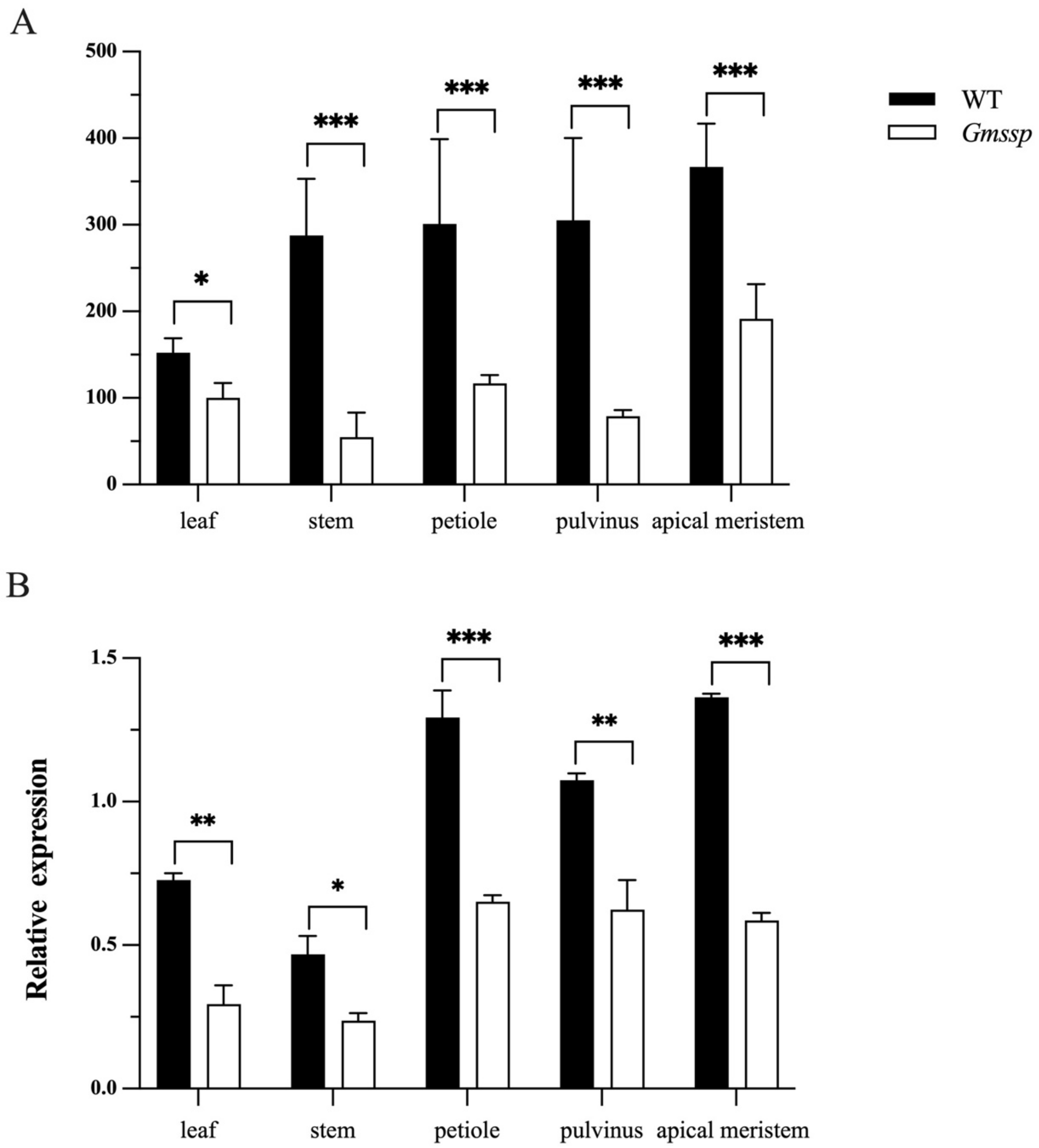

Figure S4. The expression of *Glyma.11G026400* in different tissues. (A) *Glyma.11G026400* expression was analyzed using the RNAseq-workflow pipeline. (B) Expression analysis was performed by RT-qPCR. *Tubulin 5* was used as the reference gene. Expression levels are presented as the means ± SDs from three biological replicates. *, **, and *** represent significant differences at $p < 0.05$, $p < 0.01$, and $p < 0.001$, respectively.

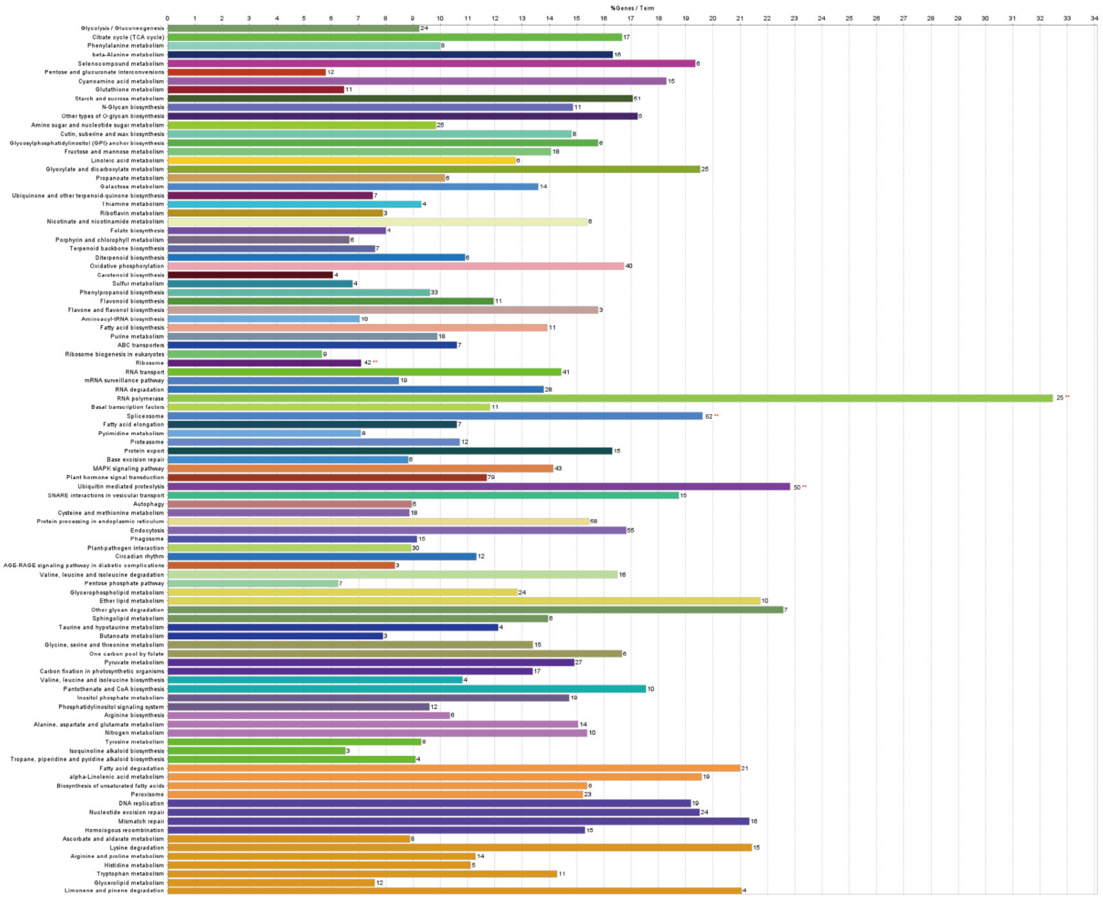

Figure S5. Enrichment analysis of GO terms and pathways among DEGs between *GmSSP* and *Gmssp* in apical stem at V4 stage using ClueGO v2.5.9. The x-axis represents the enriched GO term or pathway, y-axis is percentage of gene per term, the number of enriched genes is also indicated. These go terms or pathways with the same color next to each other indicate they are belonging to the same group according to KappaScore Grouping. ** represents statistically significance at $p < 0.01$.

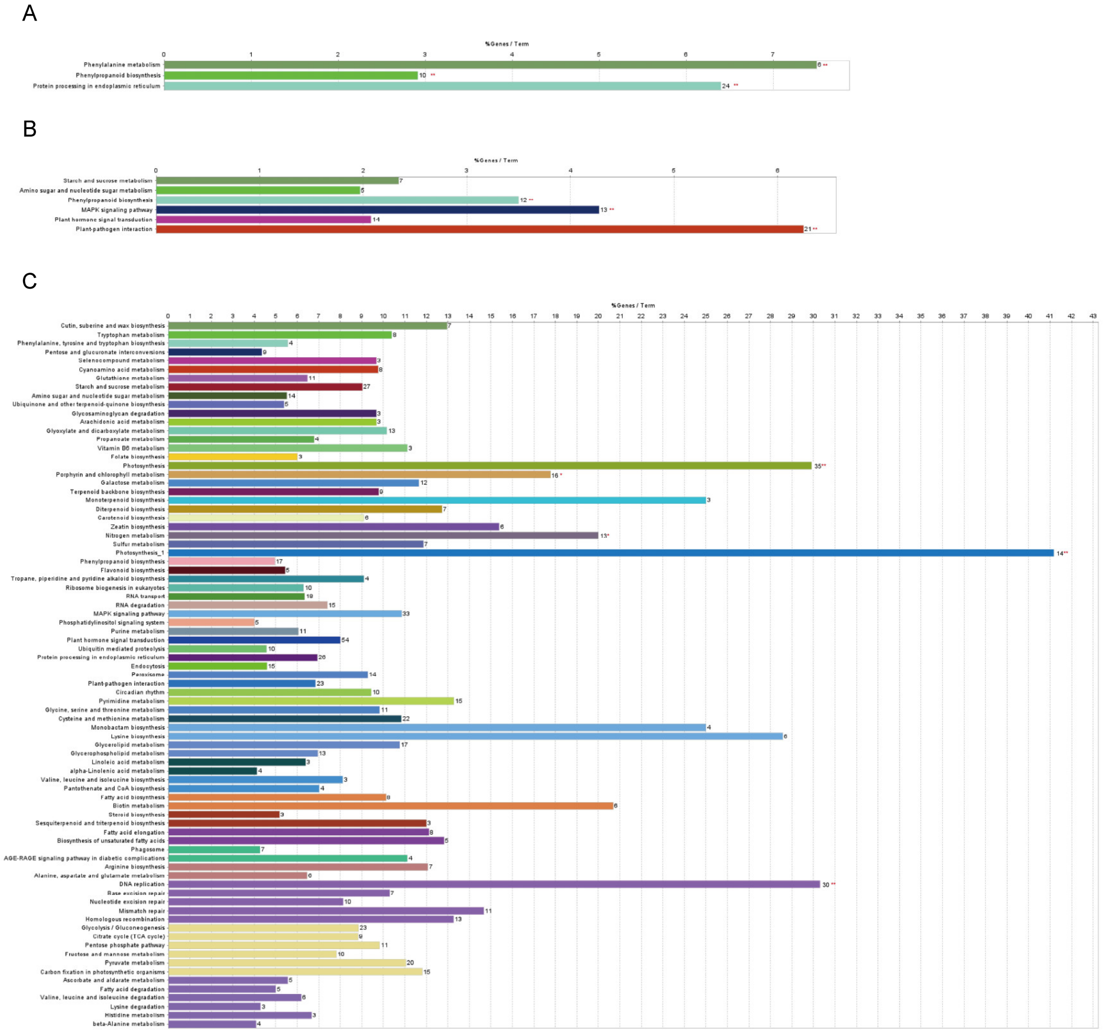

Figure S6. Enrichment analysis of GO terms and pathways among DEGs between *GmSSP* and *Gmssp* in Pulvinus (A), Stem(B) and Leaf at V4 stage(C) using ClueGO v2.5.9. The x-axis represents the enriched GO term or pathway, y-axis is percentage of gene per term, the number of enriched genes is also indicated. These go terms or pathways with the same color next to each other indicate they are belonging to the same group according to KappaScore Grouping. *, ** represents statistically significance at p <0.05 and p < 0.01, respectively.

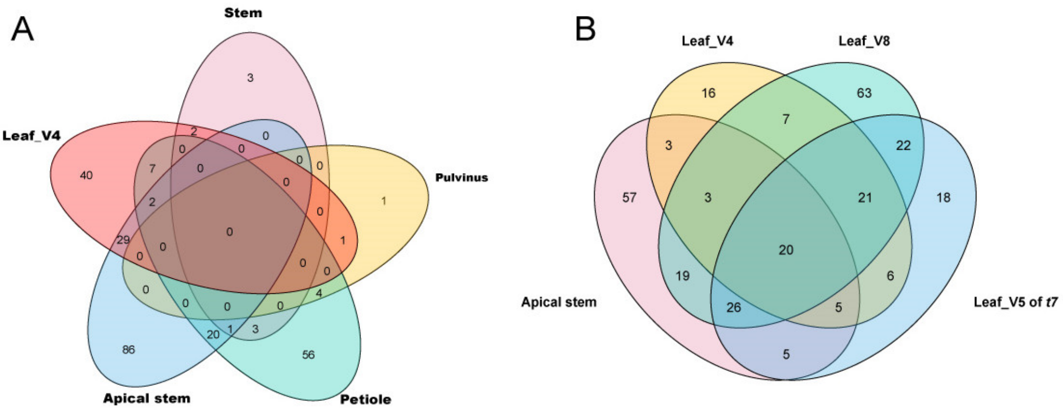

Figure S7. Venn diagrams showing the shared numbers of known pathway genes in APC-related

ubiquitin-mediated proteolysis (APC) and plant hormone transduction pathway, i.e., GA, CK, Auxin, BR. A. Shared genes between tissues of pulvinus, petiole, apical stem, leaf at V4 and stem. B. Shared genes between tissues of apical stem, leaf at V4, leaf at V8, and Leaf at V5 (*t7* and its wild-type).

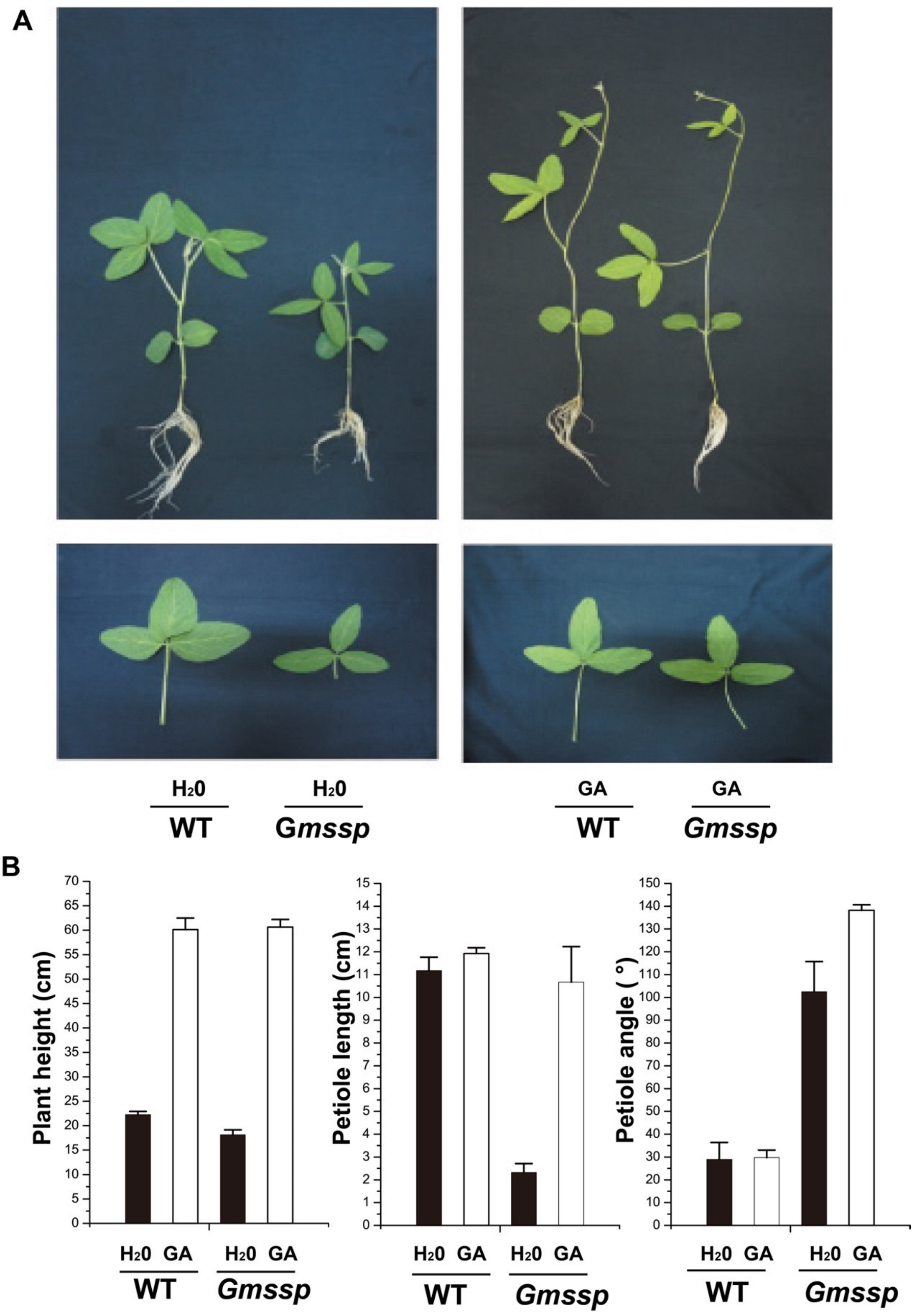

Figure S8. GA treatment could rescue the supper short petiole but not enlarged petiole angle for *Gmssp*. (A-B), the typic appearance (A), and some traits (B) on 7th day after GA_3.

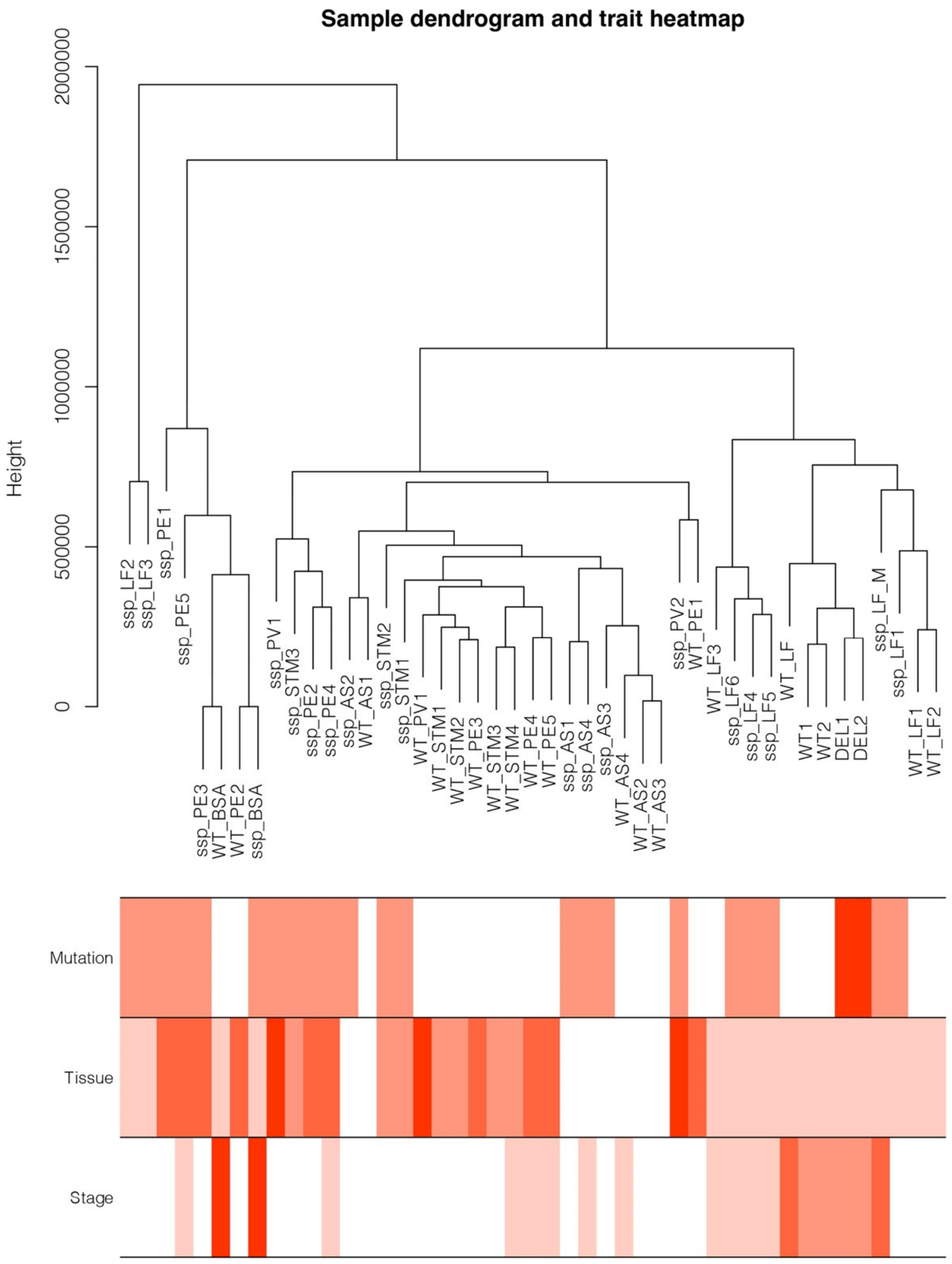

Figure S9. Sample dendrogram and trait heatmap for the transcriptom analysis and WGCNA. The basic information of samples and traits are listed in Table S9.

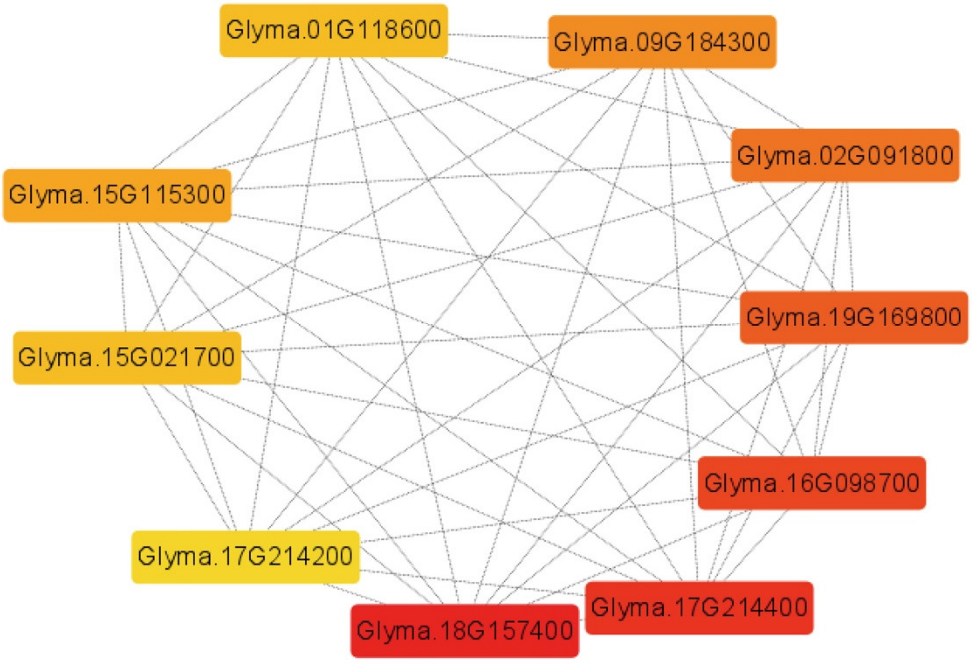

Figure S10. The putative gene network of the hub genes of in the module of MEsaddlebrown.

Supplementary Tables

Table S1. Numbers of genes manually checked on IGV using the BVF-IGV pipeline to identify the causal gene of the *Gmssp* phenotype.

Table S2. Data input for QTL mapping at the target region in chromosome 11 using QTL iciMapping (v4.1.0.0).

Table S3. Genetic variations between the *Gmssp* mutant, WT, and cultivar Hefeng 55 within the target region.

Table S4. Genes expressed in the *Gmssp* target region analyzed using the RNAseq-workflow pipeline.

Table S5. Markers used for fine-mapping and functional analysis of *Gmssp.*

Table S6. Enrichment of key pathways among different WGCNA modules.

Table S7. Functional annotation of the MEsaddlebrown module.

Table S8. Enrichment of key pathways among the DEGs in different tissues.

Table S9. Sample information for transcriptome analysis and WGCNA.
